# Lipoprotein(a) as a cardiovascular risk accelerator in primary prevention: Age-equivalent risk and event-free survival at 10 years in the UK biobank

**DOI:** 10.1016/j.ajpc.2026.101706

**Published:** 2026-06-18

**Authors:** Arturo Cesaro, Oveis Jamialahmadi, Nicola Pugliese, Valentina Flagiello, Emelie Barreby, Rosellina Margherita Mancina, Paolo Calabrò, Stefano Romeo

**Affiliations:** a Department of Translational Medical Sciences, University of Campania "Luigi Vanvitelli", Naples, Italy; b Division of Cardiology, A.O.R.N. “Sant'Anna e San Sebastiano”, Caserta, Italy; c Centre for Reproduction, Metabolism and Molecular Medicine (CeRM), Department of Medicine (H7), Karolinska Institute, Huddinge, Stockholm, Sweden; d Division of Internal Medicine and Hepatology, Department of Gastroenterology, IRCCS Humanitas Research Hospital, Rozzano, MI, Italy; e Operative Research Unit of Clinical Medicine and Hepatology, Fondazione Policlinico Universitario Campus Bio-Medico, Via Alvaro del Portillo, 200–00128 Rome, Italy; f Department of Life Science, Health, and Health Professions, Link Campus University, Rome, Italy; g Research Unit of Clinical Medicine and Hepatology, Department of Medicine and Surgery, Università Campus Bio-Medico di Roma, Rome, Italy; h Department of Endocrinology, Karolinska University Hospital, Huddinge, Stockholm, Sweden; i Clinical Nutrition Unit, Department of Medical and Surgical Science, University Magna Graecia, Catanzaro, Italy; j Department of Cardiology, Sahlgrenska University Hospital, Gothenburg, Sweden; k Department of Molecular and Clinical Medicine, University of Gothenburg, Sweden

**Keywords:** Lipoprotein(a), Atherosclerotic cardiovascular disease, Restricted mean survival time, Age-equivalent risk, Primary prevention, UK biobank

## 1 Introduction

Lipoprotein(a) [Lp(a)] is a genetically determined, causal risk factor for atherosclerotic cardiovascular disease (ASCVD), supported by concordant epidemiological and Mendelian randomization evidence [1]. Elevated concentrations affect roughly one in five adults and confer a higher risk of premature cardiovascular events [1,2]. International guidelines accordingly recommend measuring Lp(a) at least once in adulthood and treating values above 50 mg/dL (≈105-125 nmol/L) as a risk-enhancer [3]. Yet, conveying what an elevated result means for an individual is challenging. Conventional risk models rely on relative estimates, such as hazard ratios, which can feel abstract at the bedside and may understate lifetime risk in younger people [4].

Two metrics can make this burden tangible: restricted mean survival time (RMST), which expresses the absolute event-free time lost over a fixed horizon, and an age-equivalent risk, which recasts that loss as years of additional cardiovascular age [5]. Our aim is to translate the risk associated with elevated Lp(a) into absolute time lost and into an age-equivalent metric, to support clinician-patient communication and prioritization. We therefore quantified the effect of elevated Lp(a) on cardiovascular outcomes, including cardiovascular and all-cause mortality, using RMST and age-equivalent risk across age strata in a large primary-prevention cohort.

## 2 Methods

We studied unrelated participants of European ancestry in the UK Biobank, a population-based cohort that enrolled approximately 500,000 adults aged 40 to 69 years between 2006 and 2010 (application 37142; ethical approval, Northwest Multi-Centre Research Ethics Committee 21/NW/0157). We excluded participants with prevalent ASCVD at baseline (myocardial infarction, coronary revascularization, ischemic stroke, or peripheral arterial disease) and those without a baseline Lp(a) measurement. Lp(a) was measured at baseline by immunoturbidimetric assay and reported in nmol/L. We defined elevated Lp(a) as ≥105 nmol/L, the molar value reported in the 2025 ESC/EAS focused update [3] corresponding to ≥50 mg/dL, adopted as a cardiovascular risk-enhancing. Molar units are the preferred standard, and because mass-to-molar conversion factors vary (about 2 to 2.5), >50 mg/dL corresponds to roughly 105 to 125 nmol/L across sources. Outcomes were incident ASCVD (a composite of non-fatal myocardial infarction, coronary revascularization, and fatal or non-fatal ischemic stroke) plus cardiovascular death; cardiovascular death alone; and all-cause mortality. Participants were followed from baseline to the first event, death, or censoring, with cardiovascular composites censored on 31 March 2023 and mortality on 30 November 2024. We estimated hazard ratios with 95% confidence intervals (CI) using Cox proportional hazards models, adjusted for age, sex, hypertension, diabetes, low-density lipoprotein (LDL) cholesterol, and smoking. To quantify the absolute burden, we fitted flexible parametric survival models with the same covariates and computed the 10-year difference in RMST between Lp(a) groups, interpreted as the average event-free time lost over a decade; the 10-year horizon matches standard cardiovascular risk estimation. We then expressed this loss as an age-equivalent risk, defined as the additional years of chronological age that produce the same 10-year RMST decrement as elevated Lp(a). The shift was estimated within each age stratum (40–49, 50–59, and 60–69 years) and summarized as an average weighted by the number of participants at each year of age. Sensitivity analyses, including sex-stratified and competing-risk models, were performed. All analyses were performed using R (R Foundation for Statistical Computing, Vienna, Austria). Significance was set at a two-sided P < 0.05.

## 3 Results

We examined 237,729 European UK Biobank participants, of whom 36,233 (15.2%) had Lp(a) ≥105 nmol/L. The two groups were similar in age (mean 57 ± 8 years), body mass index, hypertension, and smoking. Over a median follow-up of 14 years, the composite of ASCVD plus cardiovascular death occurred in 15,855 participants (6.7%), cardiovascular death in 7,740 (3.6%), and death from any cause in 23,727 (10%). In adjusted models, Lp(a) ≥105 nmol/L was independently associated with the composite of ASCVD plus cardiovascular death (HR 1.21, 95% CI 1.16–1.26, P < 0.001), and with cardiovascular death (HR 1.11, 95% CI 1.05–1.18, P < 0.001). The signal was specific to cardiovascular outcomes: elevated Lp(a) was not associated with all-cause mortality (HR 1.01, 95% CI 0.98–1.06, P = 0.31) (Figure 1A). Estimates were materially unchanged in sex-stratified models, with no Lp(a)-by-sex interaction, and after accounting for the competing risk of non-cardiovascular death.

**Fig. 1:**
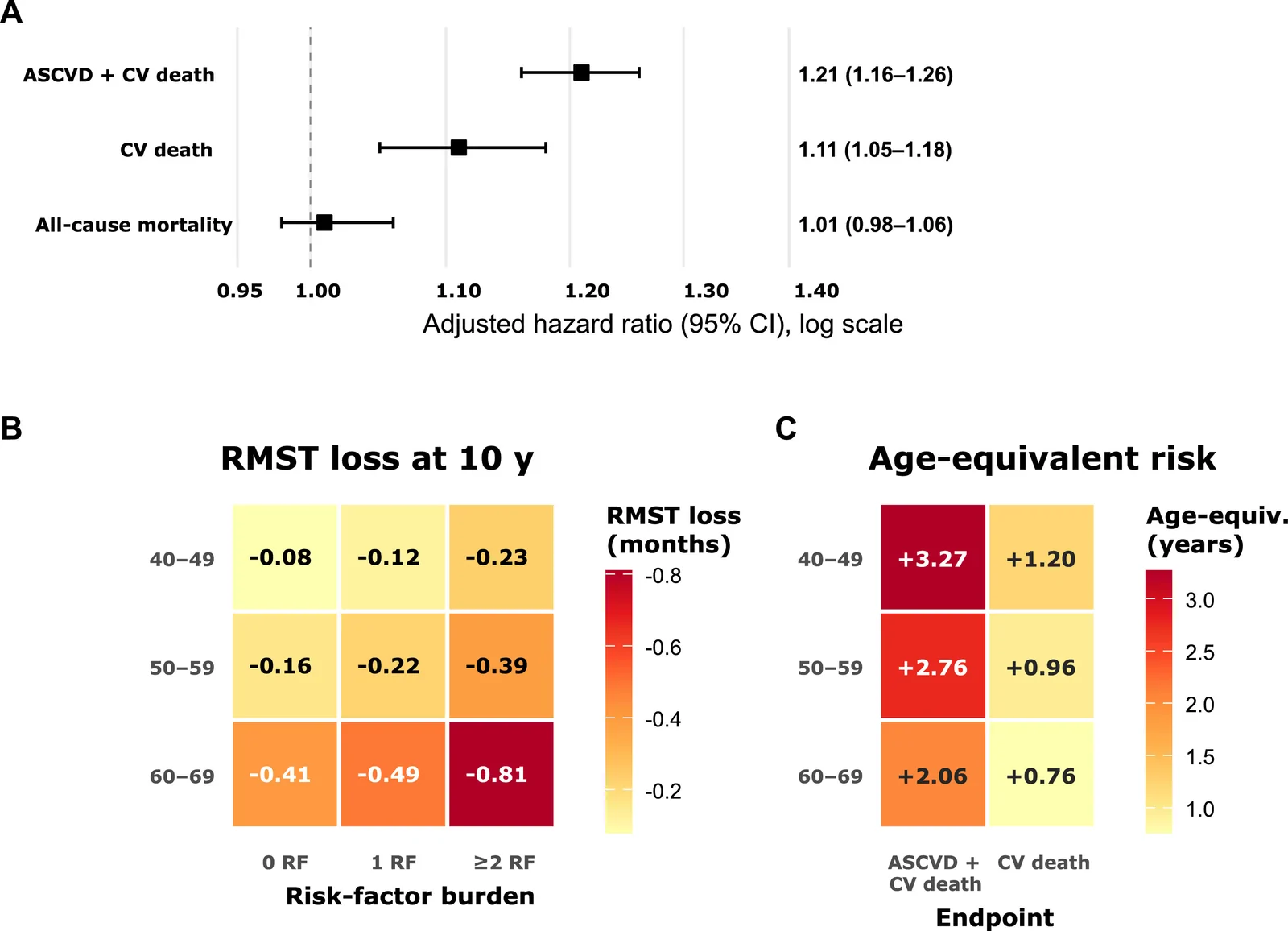
Elevated lipoprotein(a) and the 10-year burden of cardiovascular events. Data are from 237,239 UK Biobank participants of European ancestry, free of atherosclerotic cardiovascular disease (ASCVD) at baseline, comparing lipoprotein(a) [Lp(a)] ≥105 nmol/L (≈50 mg/dL) with <105 nmol/L. **(A)** Adjusted hazard ratios (95% CI) for ASCVD plus cardiovascular (CV) death, CV death, and all-cause mortality, from Cox proportional-hazards models adjusted for age, sex, hypertension, diabetes, LDL cholesterol, and smoking. The dashed line marks a hazard ratio of 1, and the x-axis is on a logarithmic scale. **(B)** Absolute loss in restricted mean survival time (RMST) free of ASCVD plus CV death over a 10-year horizon (τ = 10 years), expressed in months, from flexible parametric (Royston–Parmar) survival models using the same adjustment set. Estimates are stratified by age group (rows) and by number of traditional risk factors (columns: hypertension, diabetes, current smoking, and LDL cholesterol ≥3.0 mmol/L, counted as 0, 1, or ≥2). More negative values, shown as darker cells, denote greater event-free time lost. **(C)** Age-equivalent risk, defined as the additional years of chronological age in a person with Lp(a) <105 nmol/L that reproduce the same 10-year event-free survival as elevated Lp(a) at the index age. Values are given for ASCVD plus CV death and for CV death, stratified by age group; darker cells denote a larger age equivalent. These are point estimates from the same flexible parametric models. Color scales are panel-specific (months in B, years in C). Panels B and C share the age axis but differ on the second axis (risk-factor burden vs endpoint). ASCVD, atherosclerotic cardiovascular disease; CI, confidence interval; CV, cardiovascular; LDL, low-density lipoprotein; Lp(a), lipoprotein(a); RMST, restricted mean survival time.

To express this risk in absolute terms, we estimated the 10-year difference in RMST. Elevated Lp(a) was associated with a 10-year RMST difference of −0.38 months for ASCVD plus cardiovascular death (95% CI −0.47 to −0.30, P < 0.001), representing event-free time lost over the decade; the difference for cardiovascular death alone was smaller (−0.06 months). This absolute loss widened with age and risk-factor burden, from −0.08 months in participants aged 40 to 49 years without risk factors to −0.81 months in those aged 60 to 69 years with two or more risk factors (Figure 1B). Translated into age-equivalent terms, elevated Lp(a) at age 40 to 49 years carried a risk of ASCVD plus cardiovascular death equivalent to being 3.3 years older; the shift was 2.8 years at 50 to 59 years and 2.1 years at 60 to 69 years (Figure 1C). The absolute burden of elevated Lp(a) was therefore greatest in older, higher-risk participants, whereas its relative, age-equivalent impact was greatest in the young, in whom age-related risk has not yet accumulated.

## 4 Discussion

The main finding of this analysis is that in this primary-prevention cohort, elevated Lp(a) shortened event-free survival and carried a risk equivalent to several years of additional cardiovascular aging. Lp(a) is a well-known, established causal risk factor [6]; our contribution is a translation of that risk into absolute, intuitive terms, the event-free time lost over a decade and an age-equivalent shift, to support clinician–patient communication and prioritization. The relative effect we observed for ASCVD and CV death is consistent with prior cohort studies and Mendelian randomization studies [1,4], and the signal was cardiovascular-specific; in fact, CV events and CV death were associated with elevated Lp(a), whereas all-cause mortality was not at the guideline threshold.

Three features carry clinical implications. First, the absolute burden was concentrated among older participants with a high risk-factor burden, who lost up to 0.80 months of event-free survival over 10 years. Second, the age-equivalent shift was largest in the youngest adults (3.3 years at 40 to 49 years, narrowing to 2.1 years at 60 to 69 years); this is expected because the annual increment in age-related risk grows with age, so a Lp(a)-related effect maps onto fewer additional years later in life. The relative importance of Lp(a) is therefore greatest early, supporting measurement and risk-factor optimization before atherosclerotic burden accumulates. Third, although each individual loss is modest, across the roughly one in five adults with elevated Lp(a) it scales to meaningful population morbidity and argues for integrating Lp(a) into risk estimation rather than treating it as a binary flag.

This study has some limitations. It was confined to European-ancestry participants, and generalizability to populations with different Lp(a) distributions requires confirmation. The 10-year horizon aligns with standard risk estimation but may understate lifetime risk, particularly in younger adults with longer cumulative exposure; longer horizons would likely reveal larger absolute differences.

## 5 Conclusions

Elevated Lp(a) acts as a cardiovascular risk accelerator, shortening event-free survival and shifting cardiovascular risk by several years. RMST and age-equivalent metrics quantify the burden of Lp(a) beyond conventional hazard ratios.

## Data availability statement

The data underlying this article were provided by UK Biobank under application number 37142. UK Biobank data are available to bona fide researchers upon application at https://www.ukbiobank.ac.uk. The analytical code used in this study is available from the corresponding author upon reasonable request.

## Disclosures

A.C. has served as a member of Advisory Boards for Amgen, Novartis, Daiichi Sankyo, Boehringer Ingelheim, Novo Nordisk, Eli Lilly, Chiesi, Ultragenyx. He has also received lecture fees from Novo Nordisk and support for attending meetings from Menarini, Daiichi Sankyo, and Novartis. N.P. served as a speaker or advisor for Gilead, Gore, Astra Zeneca, AlfaSigma and Novo Nordisk. He also received travel grant from Gilead, AlfaSigma and AbbVie. In the last 5 years, S.R. received research grants from Novo Nordisk and AstraZeneca for basic science research on steatotic liver disease, and has been consulting for AstraZeneca, GSK, Celgene Corporation, Ribocure AB¸ Madrigal, Ultragenyx, Amgen, Sanofi, Wave Life Sciences, Lipigon, Novartis, Profluent, Aina, Echosense and Chiesi; declares equity from Heptabio; and is inventor on a Patent with title “Method for treating fatty liver disease”, on PSD3, US application number 17,480266 filed on 21st September 2021. All other authors declare no conflicts of interest.

## Funding

S.R. was supported by the Region Stockholm (ALF project grant, FoUI-1021801), the Swedish Cancerfonden (22 2270 Pj), the Swedish Research Council (Vetenskapsradet (VR), 2023-02079), the Swedish Heart Lung Foundation (20220334), the Novo Nordisk Distinguished Investigator Grant - Endocrinology and Metabolism (NNF23OC0082114), the Novo Nordisk Project grants in Endocrinology and Metabolism (NNF24OC0091535), and a Novo Nordisk donation to the Karolinska Institutet in connection with the professor appointment.

## CRediT authorship contribution statement

**Arturo Cesaro:** Writing – review & editing, Writing – original draft, Visualization, Methodology, Formal analysis, Conceptualization. **Oveis Jamialahmadi:** Writing – review & editing, Software, Methodology, Formal analysis, Data curation. **Nicola Pugliese:** Writing – review & editing, Validation. **Valentina Flagiello:** Writing – review & editing, Data curation. **Emelie Barreby:** Writing – review & editing, Validation. **Rosellina Margherita Mancina:** Writing – review & editing, Validation, Supervision. **Paolo Calabrò:** Writing – review & editing, Supervision. **Stefano Romeo:** Writing – review & editing, Supervision, Project administration, Conceptualization.

## Declaration of competing interest

The authors declare the following financial interests/personal relationships which may be considered as potential competing interests: Arturo Cesaro reports a relationship with Amgen Inc that includes: board membership and consulting or advisory. Arturo Cesaro reports a relationship with Novartis that includes: board membership and travel reimbursement. Arturo Cesaro reports a relationship with Daiichi Sankyo Inc that includes: consulting or advisory and travel reimbursement. Arturo Cesaro reports a relationship with Boehringer Ingelheim that includes: consulting or advisory. Arturo Cesaro reports a relationship with Novo Nordisk that includes: board membership, consulting or advisory, and travel reimbursement. Arturo Cesaro reports a relationship with Eli Lilly that includes: consulting or advisory. Arturo Cesaro reports a relationship with Ultragenyx Pharmaceutical Inc that includes: board membership and consulting or advisory. Arturo Cesaro reports a relationship with Menarini that includes: travel reimbursement. Nicola Pugliese reports a relationship with Gilead Sciences Inc that includes: speaking and lecture fees and travel reimbursement. Nicola Pugliese reports a relationship with Gore that includes: speaking and lecture fees. Nicola Pugliese reports a relationship with Alfa Sigma that includes: speaking and lecture fees and travel reimbursement. Nicola Pugliese reports a relationship with Novo Nordisk that includes: speaking and lecture fees. Stefano Romeo reports a relationship with Novo Nordisk that includes: funding grants. Stefano Romeo reports a relationship with AstraZeneca that includes: consulting or advisory and funding grants. Stefano Romeo reports a relationship with GSK that includes: consulting or advisory. Stefano Romeo reports a relationship with Celgene Corporation that includes: consulting or advisory. Stefano Romeo reports a relationship with Ribocure Pharmaceuticals AB that includes: consulting or advisory. Stefano Romeo reports a relationship with Madrigal Pharmaceuticals Inc that includes: consulting or advisory. Stefano Romeo reports a relationship with Ultragenyx Pharmaceutical Inc. that includes: consulting or advisory. Stefano Romeo reports a relationship with Amgen that includes: consulting or advisory. Stefano Romeo reports a relationship with Sanofi that includes: consulting or advisory. Stefano Romeo reports a relationship with Lipigon that includes: consulting or advisory. Stefano Romeo reports a relationship with Wave Life Sciences that includes: consulting or advisory. Stefano Romeo reports a relationship with Novartis that includes: consulting or advisory. Stefano Romeo reports a relationship with Profluent that includes: consulting or advisory. Stefano Romeo reports a relationship with Echosense that includes: consulting or advisory. Stefano Romeo reports a relationship with Chiesi that includes: consulting or advisory. Stefano Romeo reports a relationship with Heptabio that includes: equity or stocks. Stefano Romeo has patent #“Method for treating fatty liver disease”, on PSD3 licensed to US application number 17,480266 filed on 21st September 2021. If there are other authors, they declare that they have no known competing financial interests or personal relationships that could have appeared to influence the work reported in this paper.
